# Synergistic effects of plasma S100b levels and MRI‐based water exchange rate across the blood‐brain‐barrier on memory performance among older adults

**DOI:** 10.1002/alz.089815

**Published:** 2025-01-09

**Authors:** Colleen Pappas, Valentinos Zachariou, Christopher E. Bauer, Tiffany L. Sudduth, Donna M. Wilcock, Gregory A Jicha, Anika MS Hartz, Xingfeng Shao, Danny JJ Wang, Brian T Gold

**Affiliations:** ^1^ University of Kentucky, Lexington, KY USA; ^2^ University of Kentucky / Sanders‐Brown Center on Aging, Lexington, KY USA; ^3^ Indiana University School of Medicine, Stark Neurosciences Research Institute, Department of Neurology, Indianapolis, IN USA; ^4^ University of Kentucky Sanders‐Brown Center on Aging, Lexington, KY USA; ^5^ University of Kentucky College of Medicine, Lexington, KY USA; ^6^ University of Southern California, Los Angeles, CA USA; ^7^ Sanders‐Brown Center on Aging, Lexington, KY USA

## Abstract

**Background:**

Non‐invasive biofluid and MRI measures of blood‐brain‐barrier (BBB) dysfunction may aid early detection of cerebral small vessel disease (cSVD). Plasma markers of astrocytic function and injury, such as S100 calcium‐binding protein B (S100b), have gained increased attention in relation to BBB integrity and cognition. Here we explored the inter‐relationships between plasma S100b levels, an MRI measure of water exchange rate across the BBB (kw), and cognitive performance among older adults.

**Method:**

The participant sample consisted of 74 older adults without dementia recruited from the University of Kentucky Sanders Brown Center on Aging. Relationships between S100b and cognition (memory, executive function) and MRI‐based BBB water exchange rate were tested. Plasma S100b levels (pg/mL) were measured using Meso Scale Discovery R‐PLEX assay at the University of Kentucky’s CCTS Biomarker Analysis Lab. Composite scores were created for memory and executive function. A diffusion‐prepared arterial spin labeling (DP‐ASL) MRI sequence was used to estimate water exchange rate across the BBB (expressed as kw). All data (S100b, cognition, MRI) were collected within 1 year of each other. Multiple linear regression models examined the impact of plasma S100b on memory, executive function, and kw. Covariates included age, gender, and education (for cognition models only). Additionally, kw was tested as moderator of the S100b‐cognition relationships using the PROCESS macro.

**Result:**

A negative relationship was observed between S100b and memory, where higher S100b levels were associated with poorer memory performance. A similar relationship was not observed with executive function. S100b was also not associated with kw. However, there was an interaction between S100b levels and kw in the parietal lobe on memory performance such that participants with both lower parietal kw and higher S100b showed the poorest memory performance.

**Conclusion:**

Our results indicate that S100b levels are negatively associated with memory performance, but not MRI‐based BBB kw. However, higher S100b levels coupled with lower MRI‐based water exchange rate further contributed to the strong negative effects observed for memory performance. This suggests that plasma S100b and BBB kw may be different proxies of BBB function and may have synergistic negative effects on cognition.

